# Views, Barriers and Facilitators  of Policymakers, Pharmacists and Health Community Representative in Managing Unused Medicine in a Socioeconomically Diverse District in Indonesia

**DOI:** 10.12688/f1000research.177671.1

**Published:** 2026-02-24

**Authors:** Raden Aldizal Mahendra Rizkio Syamsudin, Susi Ari Kristina, Chairun Wiedyaningsih, Pauline Siew Mei Lai

**Affiliations:** 1 Pharmacy Program, Faculty of Mathematics and Natural Sciences, Universitas Garut, Garut, Indonesia; 2 Doctoral Program of Pharmacy, Gadjah Mada University Faculty of Pharmacy, Yogyakarta, Special Region of Yogyakarta, Indonesia; 3 Department of Pharmaceutical, Gadjah Mada University Faculty of Pharmacy, Yogyakarta, Special Region of Yogyakarta, Indonesia; 4 Department of Primary Care Medicine, University of Malaya Faculty of Medicine, Kuala Lumpur, Federal Territory of Kuala Lumpur, Malaysia; 5 Sir Jeffrey Cheah Sunway Medical School, Sunway University School of Medical and Life Sciences, Bandar Sunway, Selangor, Malaysia

**Keywords:** Unused medicines, Stakeholder perspectives, Qualitative study, Thematic analysis, Socio-ecological approach

## Abstract

**Background:**

Unused medicine poses a serious risk to community and environmental health. Several studies have been conducted on how patient behave however little is known about how this issue is viewed through the lens of different stakeholders.

**Objective:**

To explore the views, barriers and facilitators of policymakers, pharmacists and community-representative on community behavior in managing unused medicines in Garut Regency, Indonesia.

**Methods:**

A qualitative study was conducted with policymakers, pharmacists and community -representatives. Data were analyzed inductively using thematic analysis framework. Emergent themes were further interpreted using the Socio-Ecological approach to situate behaviors and challenges within individual, community, organizational, and policy levels.

**Results:**

Fourty one participants were recruited. Five themes were identified: (1) Storing and disposing medicines, (2) causes of unused medicines, (3) individual-level barriers including knowledge gaps and cultural beliefs, (4) structural barriers such as limited facilities, regulatory gaps, and institutional constraints, and (5) facilitators including rising awareness, supportive legal frameworks, and cross-sectoral initiatives. Mapping these findings onto the Socio-Ecological approach highlighted the interplay between patient practices, social norms, institutional resources, and policy environments.

**Conclusion:**

Stakeholders recognize that unused medicine management is shaped by multi-level factors beyond individual awareness. Effective interventions will require a comprehensive approach that integrates patient education, community engagement, health system support, and regulatory frameworks.

## Introduction

Pharmaceutical waste should become a global public health and environmental concern, with unused medicines representing a major contributor. When inappropriately consumed, especially by vulnerable populations such as children or older adults, unused medicines can lead to accidental poisoning or toxicity ( Insani et al., 2020). Expired medicine knowingly caused death to cancer patient in Yemen ( Associated Press, 2022). Improper disposal, such as burning, burying, or discarding medicines into household trash or water systems, contributes to soil and water contamination and poses risks to ecological health. This could pose serious risks as some antibiotic had already found in European river and seas ( Szymańska et al., 2019) while compound from common medicine for instance acetaminophen, β-blocker, carbamazepine, cetirizine, lidocaine, metformin, naproxen, trimethoprim or venlafaxine antidepressant had been found in every sampling site from the river at almost every continent ( Wilkinson et al., 2022). From an economic perspective, unused medicines represent wasted expenditure, not only at the household level however also for health systems and pharmacies that supply them. Estimated cost of collected medicine could be varies from 7,416 USD to 1,118,020 USD ( Wang et al., 2024).

In high-income countries, generally structured return programs and pharmaceutical waste management policies have been established. Some countries in Europe mandated by law to accept unused medicine. In contrast, many low- and middle-income countries (LMICs), lack systematic approaches at both the national and local levels resulting in improper disposal practice ( Rogowska & Zimmermann, 2022). Despite being one of the world’s most populous countries with increasing pharmaceutical consumption, Indonesia does not have a national program, regulatory framework, or budget allocation dedicated to the management of household pharmaceutical waste ( Alfian, Rendrayani, et al., 2024b). Consequently, households often rely on informal or unsafe disposal practices.

Existing research in Indonesia and comparable contexts has largely examined unused medicines from the perspective of individual patients, often focusing on medication adherence and self-medication practices ( Insani et al., 2020). Some also examined perspectives from pharmacist about their willingness as collecting agent ( Alfian et al., 2023; Alfian, Azzahra, et al., 2024a). Those work tend to emphasize individual-level knowledge and practices rather than broader structural and institutional factors. Yet, addressing unused medicines requires the involvement of multiple stakeholders, including policymakers, pharmacists, and community organizations who can influence not only household behaviours but also the systems that enable safe medicine management.

Public health frameworks emphasize that health behaviours are not solely individual choices however are shaped by factors operating across multiple levels, including individual, community, organizational, and policy environments, also entitled as Socio-ecological approach ( Golden & Earp, 2012). This socio-ecological perspective underscores the importance of examining barriers and facilitators beyond the individual, and of designing interventions that align with these layered influences. Furthermore, this system could be applied in different settings to implement transformational change in health care system, for example reducing polypharmacy incident in Canada ( Tannenbaum et al., 2017).

The present study therefore aims to explore stakeholders’ perspectives on community behaviour in managing unused medicines in Garut Regency, Indonesia. Specifically, it seeks to identify the barriers and facilitators that influence their management. By capturing insights from multiple stakeholders across government, professional, and community sectors, this study contributes to understanding how household practices intersect with systemic gaps, and provides evidence to inform the development of comprehensive strategies for pharmaceutical waste management in LMIC settings.

## Methods

### Study design

This study employed a qualitative approach with descriptive design using Focus Group Discussions (FGD) as the primary data collection method to investigate the perspective from multiple stake holder in Garut Regency. FGD was chosen to enabling multi stakeholder participation in stating their perspectives and experiences while also using each other’s in describing unused medicine management in Garut Regency ( Jakobsen et al., 2023). Qualitative research was conducted due to its multifaceted nature to explore complex phenomena, giving relevance, and add holistic perspective in understanding one issue ( Lim, 2025).

### Study setting and context

Administratively, Garut is often informally divided into three areas: northern, southern, and the central city region. Most government offices and administrative centers are located in the city area, creating disparities in access to public services, including health and environmental services, for communities in other regions ( Badan Pusat Statistik Kabupaten Garut, 2022). To address these disparities, the local Health Office operates Community Health Centers (Puskesmas) distributed across districts, supported by pharmacists who also establish private pharmacies in closer proximity to communities. In addition, the government launched the Family Welfare and Empowerment Team (Tim Penggerak Pemberdayaan dan Kesejahteraan Keluarga/TP PKK), a nationwide initiative implemented at the regency, district, and village levels. The TP PKK is composed of female volunteers who play a vital role in bridging communities and the government ( Sukmawati et al., 2025). Their responsibilities include monitoring the social and economic conditions of households and supporting health promotion initiatives at the community level. Despite these structures, no formal program or policy currently exists for the disposal of unused medicines at the household level, leaving communities without clear guidance or accessible facilities to manage pharmaceutical waste properly.

### Participant sampling and recruitment

Eligible participants were representatives from selected institutions who possessed broad knowledge of medicine management, provided pharmaceutical services, and/or had experience in community health. They were required to have at least one year of professional experience in a related field and to obtain an official recommendation from their institutional leader. Participants were excluded if they had less than one year of relevant professional experience, did not receive formal approval from their institution, were on temporary leave or not actively engaged in their professional duties during data collection, declined to provide informed consent, or had potential conflicts of interest that might compromise the integrity of the study. No prior relationship was established between the researcher and participants before the study commenced.

This study employed purposive sampling in purpose to choosing individual with relevant experience and perspective while fairly representing their institution. Purposive sampling was chosen to met convenience element in voluntary nature of all participant ( Campbell et al., 2020). Eligible participants were representatives from selected institutions who possessed broad knowledge of medicine management, provided pharmaceutical services, and/or had experience in community health. They were required to have at least one year of professional experience in a related field and to obtain an official recommendation from their institutional leader. Participants were excluded if they had less than one year of relevant professional experience, did not receive formal approval from their institution, were on temporary leave or not actively engaged in their professional duties during data collection, declined to provide informed consent, or had potential conflicts of interest that might compromise the integrity of the study. No prior relationship was established between the researcher and participants before the study commenced.

Before the FGDs were conducted, an official request letter was sent to institutional leaders along with research approval and an endorsement from National and Political Unity Agency. Each institution proposed list of participants, who then received a formal invitation. All selected participants were added to different communication group to coordinate schedules and methods of data collection, and were provided with a study overview, informed consent, a non-disclosure agreement, and a short demographic questionnaire including in Additional File 1. This process resulted in four FGDs and one separate interview with BBPOM due to scheduling constraints: Group 1 consisted of policymakers, Group 2 & 3 community pharmacists in private pharmacies and community health centers while Group 4 of PKK representatives.

### Ethical consideration and data collection

Ethical approval for this study was obtained from the Ethics Committee of the Faculty of Medicine, Public Health, and Nursing, Universitas Gadjah Mada (Number KE/FK/0965/EC/2025). All participants provided written informed consent prior to data collection. Anonymity and confidentiality were ensured by removing all personal identifiers and reporting data only at the institutional or role level.

Data collection was conducted between June and September 2025 using Focus Group Discussions (FGDs) with stakeholders from various institutions. Each FGD lasted approximately 100–130 minutes and was facilitated by a male researcher (RAMRS), a licensed pharmacist trained in qualitative methods, supported by a note-taking team. Four FGDs were held: (1) district government officials (Environmental Agency, Health Office, and Indonesian Pharmacists Association), (2) pharmacists from private pharmacies, (3) representatives of the Family Welfare and Empowerment Team (TP PKK), and (4) pharmacists from community health centers (Puskesmas). In addition, one separate interview was conducted with the Bandung National Food and Drug Authority (BBPOM) due to scheduling constraints. Two FGDs were held in person at designated meeting rooms at the University, while the other two FGDs and the individual interview were conducted via Zoom because of time and logistical barriers. The facilitator maintained a neutral stance during the discussions and was aware of his professional background as a pharmacist, which could potentially influence interpretations.

A semi-structured FGD guide was developed based on the study objectives, comprising open-ended and follow-up questions. Socio-Ecological Model (SEM) was being used as theoritical framework which conceptualises individual, interpersonal, organisational, and policy-level determinants of health behaviour. The guide explored participants’ perceptions of community practices, barriers, and facilitators related to unused medicine management across these levels. The interview questions were refined following expert consultation (SAK and CW) and pilot testing. In addition, a brief baseline demographic questionnaire captured participants’ roles, gender, institutional affiliation, and years of experience was obtained from participants prior to the discussion using a brief self-administered form.

The guide covered two main domains: (1) stakeholders’ perspectives on pharmaceutical waste management in the community and (2) perceived challenges in implementing proper management practices. The topic guide was structured according to the four levels of the Socio-Ecological Model. Examples of guiding questions are presented in Table 1 while the full FGD guide is provided in Additional File.

**Table 1.** Example of FGD topic guide based on the socio-ecological model.

SEM level	Focus area	Example questions
Individual	Current practices, knowledge, and perceptions	*“Can you describe what people usually do with leftover or expired medicines?”*
Interpersonal	Social relation to medicine waste	*“Based on your perspective, are there any factors in community such cultural, social, or economic that influence this behavior?”*
Organizational	Institutional roles and resources	*“How do health facilities or pharmacies manage unused medicines from patients?”*
Policy	Regulations and governance	*“What policies or programs that give guiding for households to dispose their unused medicines?”*
Facilitators	Opportunities and enablers	*“What encourages or supports community efforts to improve unused medicine management?”*

### Data analysis

All data were transcribed verbatim into five separate Microsoft Word files, each corresponding to one FGD or interview session and containing both the guiding questions and participants’ responses. The principal investigator verified the accuracy of the transcripts by repeatedly listening to the recordings and cross-checking them against the written text. Transcripts were shared with participants for validation and feedback evaluation. Transcripts were then imported into NVivo version 12 for coding and analysis. A thematic analysis was applied using an inductive and reflexive approach due to its flexibility ( Jakobsen et al., 2023) and guided by Braun and Clarke’s six-phase framework. First, the research team familiarized themselves with the data through repeated reading of the transcripts. Second, open coding was conducted to capture meaningful segments of text. Third, codes were organized into initial categories, which were subsequently refined into sub-themes. Fourth, emerging sub-themes were reviewed and synthesized into main themes that reflected shared concepts across the data. Fifth, these themes were critically examined and refined to ensure coherence with the dataset. Finally, each theme was clearly defined and labeled to capture its essence ( Braun & Clarke, 2006). Rather than seeking thematic saturation, data collection was designed to ensure diversity of perspectives and contextual richness across stakeholder groups. Coding and theme development were discussed collaboratively among members of the research team to enhance analytical rigor, reduce individual bias, and ensure dependability of the findings. Themes were subsequently organized using the Socio-Ecological approach to illustrate how barriers and facilitators operate across individual, community, organizational, and policy levels ( Golden & Earp, 2012). In addition, this study followed the consolidated criteria for reporting qualitative research (COREQ) and its 32-item checklist to enhance transparency and trustworthiness in the reporting process ( Tong et al., 2007).

### Rigour and trustworthiness of the study

To ensure rigour and trustworthiness, this study followed Lincoln and Guba’s four evaluative criteria. Credibility was enhanced through triangulation across diverse stakeholder groups and peer debriefing among the research team. Dependability was ensured by maintaining a detailed audit trail of methodological decisions, coding processes, and theme development. Confirmability was strengthened through collaborative analysis and transparent documentation to minimize individual bias. Transferability was supported by providing a rich description of the study context, participant characteristics, and thematic findings, enabling readers to assess the applicability of results to other settings ( Lincoln & Guba, 1985).

## Results

### Characteristics of participants

Total number of participants were 43 with 2 participants withdrawing due to scheduling issue. No repeat interviews were conducted. Most participants were adult between 25-55 years (median = 41). The majority were female (34,8%), reflecting gender composition of community organization. Most had completed tertiary-level education, and several holding postgraduate degrees, particularly among policymakers, whereas within the PKK group showed more diverse educational background. Over half of the participants had worked for more than 10 years in their respective sectors, suggesting substantial institutional experience. Details of participants’ roles, gender, and work experience are presented in Additional File 1.

### Themes and subthemes

Researcher team led by RAMRS identified four interrelated themes. The foundational theme concerns community behaviour in managing unused medicines, including practices of storage and disposal, as well as the underlying reasons shaping these practices. Themes and subthemes used in this study are presented in Table 2.

**Table 2.** Themes, and sub themes generated from the finding.

Theme	Sub theme
1. Community Behavior in Managing Unused Medicines	1. Storing medicines, even when it has expired
	2. Disposal Practices: a. Disposing into garbage b. Improper incineration c. Throwing it in drainage system
2. Community Knowledge level	3. Low community knowledge in waste management
3. Attitude Toward Unused Medicine Management	4. Community attitude: a. Reluctance to dispose of medicines due to economic considerations b. Tendency to store medicines for “just in case” purposes c. Reluctance and lack of prioritization in managing leftover medicines d. Perceptions of the impacts of leftover medicines e. Growing public awareness
4. Reasons for having unused medications at home	5. Expired medicine
	6. Patient passed away
	7. Loose packaging
	8. Non-inquisitive patients
	9. Lack of facilities to return medicine
	10. Ease of Medicines Accessibility
	11. Limitations of Regulation and Service Standards
	12. Institutional Resource Limitations
	13. Geographical Barriers
	14. Budgetary constraints of institutions
	15. Institutional authority limitations
	16. Ineffective education
	17. Facilitator for proper disposal practice

### Community behaviour in managing unused medicines

Participants described diverse behaviours related to unused medicines, including storing expired medicines at home, discarding them in household waste, burning or burying them, and even using them for non-scientific purposes. These practices illustrate how community members manage unused medicines in the absence of formal disposal facilities. Behavior and quotation are presented in Table 3.

**Table 3.** Improper community behavior in managing medicines.

Sub theme	Illustrative quotes
Storing medicines at home, even when expired	*”People often keep one or two tablets and stop using them once they feel recovered. Later, when officers visit, the medicines are neatly arranged but already covered in dust.”* (P36)
Disposing into trash	*”From the community’s side, there are some patients who behave like once there are no more symptoms, they store the medicines. Most of them get the medicines without prescriptions, especially antibiotics, and then they just throw them away into the trash.”* (P38)
Burying medicines in the ground	*“What I know is that expired medicines are first removed from their packaging. The packaging is thrown away, while the medicine itself is crushed. The final step is usually to dig a hole, bury the medicine, and cover it with soil.”* (P24)
Burning medicines	*“For the community here, medicines are still mixed with household waste. There is no waste collection system from the Environmental Agency like in the city. So, in this area, medicines are still burned together with household waste.”* (P14)
Pouring into drainage systems	*“For unused syrups, sometimes when they are no longer good, people simply pour them into the drainage system.”* (P22)
Disposing into fish ponds	*“The disposal pattern is either burning together with other household waste, or throwing them into the fish ponds located beside or behind their houses.”* (P6)
Using unused medicines for non-scientific purposes	*“I enjoy growing orchids. I take unused medicines and then put them into a spray bottle, and spray them on the plants when I see pests. Now the flowers look fresh and healthy. I believe the medicines help.”* (P30) *“A belief passed down through generations, that to make meat tender more quickly, you can add paracetamol when boiling it. But I’m not sure about the exact ratio of meat to paracetamol.”* (P25)

### Mapping of barriers and facilitators in improper medicine disposal using socio-ecological approach

**Figure 1.**
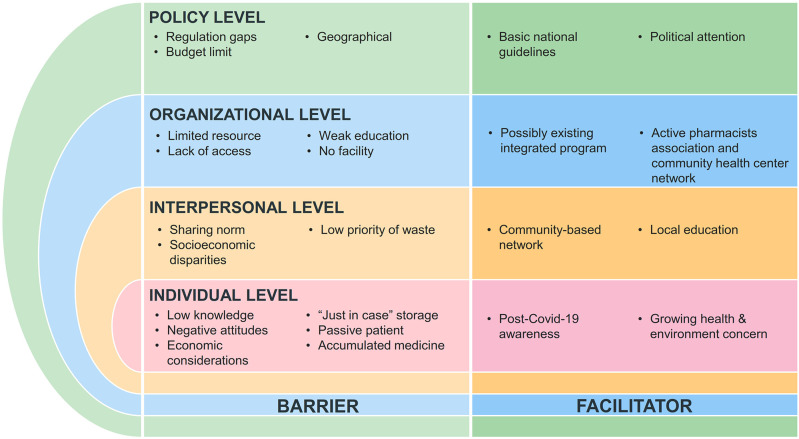
Barriers and facilitators across socio-ecological levels influencing leftover medicine management.

In addition to behavioural observations, our findings revealed multiple barriers and facilitators that influence community practices in managing unused medicines. To better illustrate the complexity of these interrelated factors, we organised them using the Socio-Ecological Model ( Golden & Earp, 2012), which distinguishes individual, interpersonal, organisational, and policy levels. Complete illustration could be found in Table 4 and Figure 1.

**Table 4.** Socio-ecological approach in mapping the barriers and facilitator of unused medicine disposal.

Level	Barrier	Facilitator
**Individual**	− Low knowledge in waste management − Negative attitudes toward leftover medicines − Economic considerations − “Just in case” practice − Passive patient − Accumulated medicine	− Growing public awareness after COVID-19. − Stronger health and environmental awareness.
**Interpersonal**	− Sharing medicines norm − Socioeconomic disparities − Low prioritization of waste management	− Experience of community-based networks that could be mobilized for household monitoring and education.
**Organizational**	− Ease of accessibility − Lack of service standards − Institutional resource limitations − Ineffective education − Absence of facilities	− Existing institutional programs that could integrated with medicine waste issues. − Readiness of Pharmacist associations and health centers to collaborate in future initiatives.
**Policy**	− Regulatory limitations − Budgetary constraints − Institutional authority limitations − Geographical barriers	− Availability of related national guidelines. − Ministerial and political attention to the issue. − Growing intersectoral collaboration initiatives.

### Individual level

At the individual level, the main issue could be affected by attitude and cultural beliefs. Many viewed discarding medicines as wasteful or financially disadvantageous, leading to the habit of storing them for “just in case” use. Passive patients also contribute to accumulated medicine in home as they tend to not question any medication they received from physicians. We could state that all of these caused by limited knowledge.

*“Sometimes people keep unused medicines therefore they or their acquaintances can use them later without buying new ones. There is also a cultural belief that throwing medicines away is wasteful.”* (P41)

*“Unused medicines are more common among adults, especially the elderly, who tend to accept whatever their medication is and accumulate them without checking expiry dates.*” (P42)

Despite these challenges, some facilitators were identified at this level. Respondents observed a gradual increase in public awareness and participation following the COVID-19 pandemic, as communities became more health-conscious.

*“From our experience, people were willing to adopt proper waste management practices, especially after COVID-19 made them more health-conscious.”* (P8)

### Interpersonal level

At the interpersonal level, social norms and family practices played a significant role in shaping behaviours. The sharing of leftover medicines among relatives or neighbours was reported as a common practice, particularly in lower-income communities. They also viewed medicine disposal as minor issue and possibly didn’t want to contribute financially as in current conditions people often rejected to pay waste transporting fee. Participant from Health Office once mentioned their experiences in finding Digoxin being sold in small kiosk (warung) due to seller’s ignorance.

*“People often give unused medicines to their family or friends if they have similar symptoms, rather than disposing of them.”* (P36)

### Organizational level

At the organizational level, several structural and institutional barriers emerged. Respondents consistently mentioned the absence of standardized procedures and limited disposal facilities. Health facilities also faced workforce and resource constraints that limited their ability to conduct consistent education and outreach. Mostly participants from Health Office and Community Health Center stated their problem in not having enough resources for even standard pharmaceutical services.

*“Even when people are aware that medicines must be properly managed, they still ask, ‘Where should this be taken?’ because the facilities are limited.”* (P5)

### Policy level

At the policy level, barriers related to regulation, coordination, and budget allocation were dominant. Respondents emphasized the absence of clear national or local regulations governing household pharmaceutical waste, leading to uncertainty among implementers. Fragmented institutional responsibilities also hampered effective management as no special taskforce was appointed to be in charge.

*“Currently, no mandatory regulations govern this issue. If such policies existed, they would come with programs and budgets.”* (P42)

Nevertheless, participants recognized several emerging facilitators at this level, including the existence of national guidelines and the growing political attention such as from The Coordinating Ministry for Human Development and Cultural Affairs or The Regent toward pharmaceutical waste.

*“The Directorate General of Pharmaceutical Services issued Decree No. HK 02031708/2021, which provides household-level guidelines for managing expired and damaged medicines.”* (P8)

Intersectoral initiatives and community-based programs such as Climate Village (Kampung Iklim), or Eco-friendly Village (Kangraling) by Environmental Agency, and Pharmacists Go to Village (Sonagar Mapay ka Lembur) by the Pharmacists Association were viewed as potential platforms to integrate unused medicine management into broader environmental and health policies.

## Discussion

This study explored how stakeholders from different institutions view unused medicines as an issue in community. We identified their perspectives on community practices, barriers and facilitator related to unused medicines management in Garut regency. By using socio-ecological approach as an analytical framework, we can explain that community behaviour in managing unused medicine could be shaped by various things worked at the individual, interpersional, organizational, and policy level.

### Community behaviours and household practices

The storage and unsafe disposal of unused medicines were constantly mentioned by stakeholders, mainly those whose directly interact with the people. Many community members most likely to keeping medicines at home, sometimes beyond expiry, or discarding them into household trash, open burning, or even throwing it into fish ponds. These practices could lead to more environmental hazard. Burning medicine in low temperatures could produce toxic air pollutant caused by the halogens structure in the structure and leave ashes as the product of heavy metals contained by some dietary supplements such as iron, zinc, manganese etc ( Alnahas et al., 2020). Recent study in China told us that at least 62 active pharmaceutical ingredients were detected in leachate sample taken from waste landfill slide). In addition, unused medicines were sometimes side-tracked for non-medical, likewise, non scientific purposes, such as tenderizing meat or spraying plants. Though acetamoniphen is chemically probable to be used as meat tenderizer, these action could be led to hazardous impact on liver, kidney and reproductive general physiological signaling ( Ezugwu et al., 2023). No study had been conducted in evaluate potential risk of human exposure to medicine-watered plant, however recent finding in several plants exposed to an solution containing pharmaceutical ingredient explain how the chemical substances could be potentially taken up to shoots and roots of edible plants ( Tanoue et al., 2012). Leaving us in the risk of accidental accumulation of API in human bodies.

Similar studies in low and middle-income countries, which have documented similar behaviours driven by limited awareness of environmental risks and weak disposal infrastructure ( Mohammed & Al-Hamadani, 2023; Mostafanejad et al., 2025). In similar contexts where pharmaceutical return programs are present, households in high income country showing same behavior i.e throwing into bin or flush it down to the sink ( Kelly et al., 2018). More finding could be done to explain what things could influence those behaviours.

### Individual level barriers to proper disposal of unused medicine

Barriers at the individual level reflected both knowledge and attitudinal dimensions. Low community knowledge about waste management in general, combined with limited understanding of pharmaceutical hazards, reduced the likelihood of good attitude and possibly safe practices ( Ayele & Mamu, 2018; Kusuma et al., 2023; Sim et al., 2018). While representatives from the community stated their lack of knowledge in safely disposing of medicine, stakeholders from local health and environment sector also mentioned poor obedience showed by the communities in the smalest scale of waste management, sorting the trash. Public in Asian is more likely to do waste sortation when they have perception of environmental risk ( Cai et al., 2024). Furthermore, medicines were often regarded as valuable commodities, leading to reluctance in discarding them. Economic considerations reinforced this tendency: households preferred to keep medicines for future use to avoid costs, reflecting cultural perceptions of medicines as resources not to be wasted. These attitude often led to practice of medicine reuse as some studies found it cost saving, especially in more expensive medicine ( Alhamad et al., 2018).

Accumulated medicine could be directly caused by expired products, medicines left behind after patient death, and drugs dispensed in loose packaging without clear labeling. Demised patient potentially left half of their medication unused and could be end in their family not using it anymore ( Ekedahl, 2006). Loose packaging led to inadequate label or unclear instruction and could end with patient confusion and tend to not using the medicine. These phenomenon observed in Srilanka which inadequate label and packaging mostly happened to medicine obtained from public sector compared to private sector ( Athuraliya et al., 2016). Passive patient attitudes, particularly among older adults, contributed to stockpiling. Active patient which had known more about their medication tend to had better management of their medication once discharged from hospital care ( McTier et al., 2015). This highlights the need for preventive strategies such as knowledge sharing, rational prescribing and controlled dispensing.

### Interpersonal level barriers to proper disposal of unused medicine

Our findings empashized social aspect, like norms and socioeconomic disparities, shape the management of unused medicines within households and communities. The practice of sharing medicines was frequently reported, highlight cultural norms where medicines are perceived as communal valuable resources rather than personal prescriptions. This practice could be seen as a positive thing and participant often have stated that this was an act of justice in the way of saving cost and improve their social relationship ( Beyene et al., 2016). However it raise some concern regarding increasing risk of drug resistance ( Dimitrov et al., 2012) or kidney failure ( Makówka et al., 2015) in population. Similar patterns have been documented in both Asian (S. Song et al., 2022) and African contexts ( Obol et al., 2018), where medicine reuse and sharing are normalized.

Furthermore, participant from higher economic status mentioned that some of their worker often asked for their leftover medicines to be taken home. This socioeconomic discrepancies explained as lower-income groups that rely on leftover medicines from others to reduce healthcare costs as also seen in methadone maintenance patient ( Caviness et al., 2013). Finally, proper disposal of unused medicines is rarely prioritized, as it is perceived as a burden without immediate benefits to households. Some participant mentioned that this act of proper disposal didn’t benefit them, difficult to implement or just have no motivation in doing therefore. The lack of tangible incentives, coupled with limited awareness of environmental and health risks, leads to low community engagement in safe disposal practices. Study in middle income country also mentioned incorrect practice of disposal when no incentives provided ( Lago et al., 2022). Together, these barriers illustrate how economic constraints and cultural norms strengthen improper practices at the community level, highlighting the need for both educational interventions and system-level solutions to provide more accessible and acceptable disposal pathways.

### Organizational level barriers to proper disposal of unused medicine

At the organizational level, several systemic barriers were identified that could be postponing effective management of unused medicines. Main point is medicines are too easily accessible, as they are frequently sold in small shops without prescriptions, while overprescribing practices within health facilities further contribute to accumulation at the household level. Recent study in same country highlighted antibiotic availability in three quarters of roadside stall (kiosk) asked. Some dispensed as repackaged blister strip resulting to out of pharmacopeia standard in 18% of the sample ( Hadi et al., 2010). This practice in LMIC could be caused by the business orientation, customers’ demand, lack of regulations; and staff’s lack of knowledge and poor attitudes about medicine use ( Belachew et al., 2021). Some study also mentioned high overprescription practices by physicians however in contrast, participant showed good knowledge and attitudes toward appropriate antibiotic use ( Amin et al., 2022). This reflects external factor such as weak enforcement of dispensing regulations and creates a surplus of medicines in the community. Heightening this issue is the lack of standardized service protocols which health centers, pharmacies, and professional associations such as the Indonesian Pharmacists Association reported uncertainty in handling unused medicines due to the absence of clear SOPs. Similar to recent finding that reported that there were no regulation in handling household pharmaceutical waste, only addressing waste from healthcare institution or pharmacies ( Alfian, Azzahra, et al., 2024a) and confusion due to low awareness of the guideline and no reported training on how to managing medication waste ( Alfian, Rendrayani, et al., 2024b). This also influenced by the absence of formal return facilities. It left households without viable options for safe disposal. Studies conducted in Portland compared communities in place with returned medicine dropbox showed higher behavior to safely dispose their medicine compared to another without ( Ehrhart et al., 2020). Institutional capacity also plays a critical role, as limited numbers of health workers and pharmacists are often required to prioritize clinical service delivery, leaving pharmaceutical waste education and monitoring under-addressed. Although educational initiatives such as “Counseling, Information and Education” (KIE), “Lets Throw Medication Waste” (ABSO), and “Smart Medicine Use Movement for Community” (Gema Cermat) have been introduced to improve public awareness, their reach and impact remain limited, with many community members reporting minimal exposure or comprehension. Some studies suggest that even the methods given (didactic) was effective in increasing public knowledge ( Kristina et al., 2021), some program like ABSO still not too recognized in local level ( Wahyudi & Kristina, 2022). Those finding leaves message to address more in medicine disposal using available program. Together, these organizational challenges reveal how gaps in accessibility control, professional guidance, human resources, and health education may be contribute to unsafe community practices, emphasizing the need for stronger institutional frameworks and coordinated efforts to integrate medicine disposal into routine healthcare delivery.

### Policy level barriers to proper disposal of unused medicine

At the policy and structural level, the management of household pharmaceutical waste is constrained by several systemic limitations. Regulatory frameworks remain unclear, as no comprehensive national or local regulations specifically address the disposal of unused medicines at the household level. This regulatory gap translates into the absence of formal mandates or accountability structures, leaving frontline institutions uncertain about their roles. This underlined similar patterns in Asean countries where there were still lack of pharmaceutical waste management even in Indonesia, Malaysia, Philippines, Singapore, Thailand or Vietnam ( Sapkota & Pariatamby, 2025). It still unclear who is responsible for its governance wether it is government or private sector ( Ravinetto et al., 2025).

Financial constraints further exacerbate the problem, since no specific budget allocation has been made for pharmaceutical waste management outside of healthcare facilities, making it difficult for local authorities to design and sustain interventions. OECD Report (2022) mentioning 34 countries with pharmaceutical waste management system. Particularly, no LMIC countries mentioned in this report. Some of those countries were funded by government wether it is by ministry, district board or office. Some of them by private sector through extended producer responsibilities scheme, and some of them funded by healthcare facilities it self ( Organisation for Economic Co-operation and Development, 2022). This could raise option to openning up into some scheme in managing pharmaceutical waste. In addition, institutional authority is fragmented: responsibilities are distributed across the Health Office, Environmental Agency, and professional associations, yet without clear coordination mechanisms, their efforts often overlap or remain incomplete. For example the association clearly mentioned that their authority is limited to their member hence the agency also mention their lack of human resources. Similar cases happens in local setting highlighting limited resources from government or community health center to solve medical waste problem. In addition they add community medical waste bank program to help processing the waste in their area ( Syafriani et al., 2021). Therefore, collaborative governance modelling runs in China give a simulation of participation rate through different models. Collaboration between government agency, enterprises and resident participation could increase participation for each parties in handling waste (W. Song et al., 2025). This could call for more community involvement in handling this issue together.

Finally, geographical barriers pose practical challenges, as Garut’s wide and diverse terrain hinders consistent outreach, education, and monitoring, particularly in rural and remote communities. These disparities could resulting in poor waste management as people in rural area less likely to have an access to improved sanitation compared to urban areas ( Irianti & Prasetyoputra, 2021). This could ensuing inequalities in accessing pharmaceutical services in region with low number of pharmacies ( Ismail & Setyawan, 2025). These structural weaknesses highlight the urgent need for integrated policy frameworks, designated funding streams, and cross-sectoral collaboration to ensure that household pharmaceutical waste is managed in a safe, equitable, and sustainable manner.

### Facilitators and opportunities for intervention

Despite these challenges, participants highlighted several facilitators that could support future interventions. Increased public awareness, particularly after COVID-19, suggested growing receptiveness to safe practices. This phenomenon could be related to growing awareness influenced by cultural, social, or biological norm post COVID-19 ( Raesi et al., 2024). Study in Asia unveiled that people tend to have adequate knowledge and collective awareness regarding medical waste because they experienced living with medical equipment i.e gloves or masks and medicine in their everyday life ( Alomari et al., 2021). The existence of national guidelines provided a potential legal foundation, although their local implementation remained limited. Ongoing programs led by PKK volunteers, environmental initiatives such as Kampung Iklim, and pharmacist-led campaigns demonstrated avenues for integrating unused medicine management into existing community and institutional platforms. These facilitators reflect opportunities for multi-sectoral collaboration, aligning with calls for integrated approaches to pharmaceutical waste management. Attempt to collaborate in increasing medication safety had been launched in Kuwait. Their take-back campaign implemented through coordination and communication among several stakeholders i.e primary care provider, drug regulation officer, medical waste management, environmental protection agency, academia, international organizations, non-governmental support, and community member ( Abahussain et al., 2024).

### Positioning findings within a socio-ecological perspective

Although the themes were derived inductively, their interconnections can be mapped onto different levels of influence, consistent with the socio-ecological model. Household behaviours reflect individual knowledge, attitudes, and economic considerations. These are embedded within community norms of sharing and storing medicines, constrained further by institutional gaps in regulation, resources, and infrastructure, and shaped ultimately by the broader policy environment. This layered understanding underscores the importance of multi-level interventions. Awareness campaigns at the household level are necessary however insufficient without parallel investments in institutional capacity and regulatory enforcement. Likewise, community-based monitoring initiatives, such as PKK household visits, can only be effective if supported by policy frameworks and resource allocation.

Taken together, these findings suggest that addressing unused medicine management in Garut requires a comprehensive strategy that engages actors across multiple levels. While the absence of local policies and return systems currently hinders safe practices, the presence of community networks, national guidelines, and increasing public awareness provides a foundation for developing context-specific, multi-level interventions.

## Conclusion

This study demonstrates how stakeholders perceive management of unused medicines in Garut Regency is shaped by complex and interrelated factors at the individual, community, organizational, and policy levels. Stakeholders highlighted unsafe household medicine practices, cultural norms of medicine reuse, weak institutional protocols, and fragmented regulatory responsibilities as key barriers. At the same time, facilitators such as increasing public awareness, existing national guidelines, and community-led initiatives present important opportunities for improvement. These findings emphasize that interventions cannot rely solely on patient education however must adopt a comprehensive and coordinated strategy that engages households, health professionals, local authorities, and regulatory bodies. Strengthening cross-sectoral collaboration, clarifying institutional roles, and ensuring dedicated resources are essential steps to build sustainable systems for safe pharmaceutical waste management.

### Strength and limitation

A strength of this study lies in its diverse stakeholders, providing a multi-layered understanding of the issue. The qualitative approach and separated FGDs enabled the identification of context-specific barriers and facilitators that may not emerge in survey-based studies in each stakeholders context. However, several limitations should be acknowledged. First, the study was conducted in a single regency, which may limit generalizability across different regions of Indonesia. Second, participant perspectives may reflect institutional view points more strongly than those of the wider community. Finally, while the socio-ecological approach offers broad explanatory power, it may oversimplify interactions between levels and does not measure the relative magnitude of each factor.

## Ethical considerations

Ethical approval for this study was obtained from the Ethics Committee of the Faculty of Medicine, Public Health, and Nursing, Universitas Gadjah Mada (Number KE/FK/0965/EC/2025). All participants provided written informed consent prior to data collection. Anonymity and confidentiality were ensured by removing all personal identifiers and reporting data only at the institutional or role level.

## Data Availability

Figshare: Underlying data for Views, Barriers and Facilitators of Policymakers, Pharmacists and Health Community Representative in Managing Unused Medicine in a Socioeconomically Diverse District in Indonesia. https://doi.org/10.6084/m9.figshare.31015444 ( Syamsudin et al., 2026) This project contains participant demographic characteristics. Access to the interview transcripts is restricted due to ethical and confidentiality considerations. Researchers may request access by contacting the author at aldizal@uniga.ac.id Data are available under the terms of the Creative Commons Attribution 4.0 International license (CC BY 4.0). Figshare: Extended data for Views, Barriers and Facilitators of Policymakers, Pharmacists and Health Community Representative in Managing Unused Medicine in a Socioeconomically Diverse District in Indonesia. https://doi.org/10.6084/m9.figshare.31015444 ( Syamsudin et al., 2026). This project contains the following extended data:
1. FGD Guide (Guide used to facilitate focus group discussions and interviews). 2. Thematic coding framework (Example of the coding framework developed during qualitative analysis). 3. Informed consent template (Template of the informed consent form provided to participants). Data are available under the terms of the Creative Commons Attribution 4.0 International license (CC BY 4.0). Repository: COREQ checklist and for ‘Views, Barriers and Facilitators of Policymakers, Pharmacists and Health Community Representative in Managing Unused Medicine in a Socioeconomically Diverse District in Indonesia’. https://doi.org/10.6084/m9.figshare.31015321.
