# Views, Barriers and Facilitators  of Policymakers, Pharmacists and Community Health Representative in Managing Unused Medicine in a Socioeconomically Diverse District in Indonesia

**DOI:** 10.12688/f1000research.177671.2

**Published:** 2026-05-04

**Authors:** Raden Aldizal Mahendra Rizkio Syamsudin, Susi Ari Kristina, Chairun Wiedyaningsih, Pauline Siew Mei Lai

**Affiliations:** 1 Pharmacy Program, Faculty of Mathematics and Natural Sciences, Universitas Garut, Garut, Indonesia; 2 Doctoral Program of Pharmacy, Gadjah Mada University Faculty of Pharmacy, Yogyakarta, Special Region of Yogyakarta, Indonesia; 3 Department of Pharmaceutical, Gadjah Mada University Faculty of Pharmacy, Yogyakarta, Special Region of Yogyakarta, Indonesia; 4 Department of Primary Care Medicine, University of Malaya Faculty of Medicine, Kuala Lumpur, Federal Territory of Kuala Lumpur, Malaysia; 5 Sir Jeffrey Cheah Sunway Medical School, Sunway University School of Medical and Life Sciences, Bandar Sunway, Selangor, Malaysia

**Keywords:** Unused medicines, Stakeholder perspectives, Qualitative study, Thematic analysis, Socio-ecological approach

## Abstract

**Background:**

Unused medicine poses a serious risk to community and environmental health. Several studies have been conducted on how patient behave. However little is known about how this issue is viewed through the lens of different stakeholders.

**Objective:**

To explore the views, barriers and facilitators of policymakers, pharmacists and community-representative on community behavior in managing unused medicines in Garut Regency, Indonesia.

**Methods:**

A qualitative study was conducted with policymakers, pharmacists and community -representatives. Data were analyzed inductively using thematic analysis framework. Emergent themes were further interpreted using the Socio-Ecological approach to situate behaviors and challenges within individual, community, organizational, and policy levels.

**Results:**

Fourty one participants were recruited. Five themes were identified: (1) Storing and disposing medicines, (2) causes of unused medicines, (3) individual-level barriers including knowledge gaps and cultural beliefs, (4) structural barriers such as limited facilities, regulatory gaps, and institutional constraints, and (5) facilitators including rising awareness, supportive legal frameworks, and cross-sectoral initiatives. Mapping these findings onto the Socio-Ecological approach highlighted the interplay between patient practices, social norms, institutional resources, and policy environments.

**Conclusion:**

Stakeholders recognize that unused medicine management is shaped by multi-level factors beyond individual awareness. Effective interventions will require a comprehensive approach that integrates patient education, community engagement, health system support, and regulatory frameworks.

## Introduction

Pharmaceutical waste is a growing a global health and environmental concern, with unused medicines representing a major contributor. When used inappropriately, particularly among vulnerable populations such as children and older adults, unused medicines—including expired products—can lead to accidental poisoning or toxicity, with documented cases of fatal outcomes among cancer patients ( Insani et al., 2020; Associated Press, 2022). Improper disposal, such as burning, burying, or discarding medicines into household trash or water systems, contributes to soil and water contamination and poses risks to ecological health. For example, antibiotics have been detected in European rivers and seas ( Szymańska et al., 2019) while residues of commonly used medicine, such as acetaminophen, carbamazepine, cetirizine, or trimethoprim had been found in every sampling site from the river at almost every continent ( Wilkinson et al., 2022). Economically, unused medicines represent substansial waste for household and health care system, with estimated loss ranging from 7,416 USD to 1,118,020 USD ( Wang et al., 2024).

In high-income countries, structured medicine return programs and regulatory frameworks for pharmaceutical waste management are well established, with some European countries mandating pharmacies to accept unused medicines. In contrast, many low- and middle-income countries (LMICs) lack systematic approaches at both national and local levels, resulting in widespread improper disposal practices ( Rogowska & Zimmermann, 2022). Despite being one of the world’s most populous countries with increasing pharmaceutical consumption, Indonesia lacks a dedicated regulatory and financing framework for consumer-level medicine take-back programmes ( Alfian, Rendrayani, et al., 2024b). This situation leaves household pharmaceutical waste ambiguously governed, despite the existence of hazardous waste classifications and non-mandatory national guidelines. As a result, responsibility for managing unused medicines remains unclear across stakeholders ( Alfian, Azzahra, et al., 2024a; Indonesia Ministry of Health, 2021; Subadi, 2022). With growing environmental concerns, understanding how unused medicines are managed has become increasingly urgent, particularly in settings where regulatory systems remain underdeveloped.

Existing research in Indonesia and comparable contexts has largely examined unused medicines from the perspective of individual patients, often focusing on medication adherence and self-medication practices ( Insani et al., 2020). Some also examined perspectives from pharmacists about their willingness as collecting agents ( Alfian et al., 2023; Alfian, Azzahra, et al., 2024a). Those works tend to emphasize individual-level knowledge and practices rather than broader structural and institutional factors. Addressing these challenges requires a multi-level perspective. A socio-ecological framework recognizes that health behaviours are shaped by interacting influences at the individual, community, organizational, and policy levels ( Golden & Earp, 2012). This framework highlights the need to address barriers and facilitators beyond the individual and has been applied to drive system-level change, such as reducing polypharmacy in Canada ( Tannenbaum et al., 2017). However, to our knowledge, no study has examined policymakers, pharmacists, and community representatives simultaneously within a single qualitative framework in the context of of Indonesia and similar UMIC settings.

This study aims to examine the perspectives of policymakers, pharmacists, and community representatives regarding the management of unused medicines in Garut Regency, Indonesia, using a socio-ecological framework. It seeks to identify barriers and facilitators operating across multiple levels that influence household medicine management practices. By integrating insights from diverse stakeholder groups, this study provides a comprehensive understanding of how individual behaviours interact with broader institutional and policy environments. The findings offer context-specific evidence to inform future policy and intervention strategies, while acknowledging that transferability to other settings requires careful consideration of local contexts.

## Methods

### Study design

This study employed a qualitative descriptive design using Focus Group Discussions (FGDs) as the primary data collection method to explore perspectives from multiple stakeholders in Garut Regency. FGDs were chosen to enable interactive discussions, allowing participants to share and build upon each other’s experiences in relation to unused medicine management ( Jakobsen et al., 2023). A qualitative approach was considered appropriate to explore the complexity of the phenomenon and to provide a holistic understanding of the issue ( Lim, 2025).

### Study setting and context

Administratively, Garut is often informally divided into three areas: northern, southern, and the central city region. Most government offices and administrative centers are located in the city area, creating disparities in access to public services, including health and environmental services, for communities in other regions ( Badan Pusat Statistik Kabupaten Garut, 2022). To address these disparities, the local Health Office operates Community Health Centers (Puskesmas) distributed across districts, supported by pharmacists who also establish private pharmacies in closer proximity to communities. In addition, the government launched the Family Welfare and Empowerment Team (Tim Penggerak Pemberdayaan dan Kesejahteraan Keluarga/TP PKK), a nationwide initiative implemented at the regency, district, and village levels. The TP PKK is composed of female volunteers who play a vital role in bridging communities and the government ( Sukmawati et al., 2025). Their responsibilities include monitoring the social and economic conditions of households and supporting health promotion initiatives at the community level. Despite these structures, no formal program or policy currently exists for the disposal of unused medicines at the household level, leaving communities without clear guidance or accessible facilities to manage pharmaceutical waste properly.

### Participants sampling and recruitment

This study employed purposive sampling to recruit participants with relevant experience and perspectives across stakeholder groups. Purposive sampling was used to recruit participants with their relevant expertise and nature perspectives across stakeholder groups ( Campbell et al., 2020). Eligible participants were representatives from selected institutions who possessed broad knowledge of medicine management, provided pharmaceutical services, and/or had experience in community health. They were required to have at least one year of professional experience in a related field and to obtain an official recommendation from their institutional leader. Participants were excluded if they had less than one year of relevant professional experience, did not receive formal approval from their institution, were on temporary leave or not actively engaged in their professional duties during data collection, declined to provide informed consent, or had potential conflicts of interest that might compromise the integrity of the study. No prior relationship was established between the researcher and participants before the study commenced.

Before the FGDs were conducted, an official request letter was sent to institutional leaders along with research approval and an endorsement from the National and Political Unity Agency. Each institution proposed a list of participants, who then received a formal invitation. All selected participants were added to different WhatsApp communication groups to coordinate schedules and methods for the data collection. Each group was then provided with a study overview, informed consent, a non-disclosure agreement, and a short demographic questionnaire, including in Additional File 1. This process resulted in four FGDs and one separate interview with BBPOM due to scheduling constraints: Group 1 consisted of policymakers, Group 2 & 3 community pharmacists in private pharmacies and community health centers, while Group 4 consisted of PKK representatives.

### Ethical considerations

Ethical approval for this study was obtained from the Ethics Committee of the Faculty of Medicine, Public Health, and Nursing, Universitas Gadjah Mada (Number KE/FK/0965/EC/2025). All participants provided written informed consent prior to data collection. Anonymity and confidentiality were ensured by removing all personal identifiers and reporting data only at the institutional or role level.

### Data collection

Data collection was conducted between June and September 2025 using Focus Group Discussions (FGDs) with stakeholders from various institutions. Each FGD lasted approximately 100–130 minutes and was facilitated by a male researcher (RAMRS), a licensed pharmacist trained in qualitative methods, supported by a note-taking team. Four FGDs were held: (1) district government officials (the Environmental Agency, the Health Office, and Indonesian Pharmacists Association), (2) pharmacists from private pharmacies, (3) representatives of the Family Welfare and Empowerment Team (TP PKK), and (4) pharmacists from community health centers (Puskesmas). In addition, one separate interview was conducted with the Bandung National Food and Drug Authority (BBPOM) due to scheduling constraints. Two FGDs were held in person at designated meeting rooms at the University, while the other two FGDs and the individual interview were conducted via Zoom because of time and logistical barriers. While both in-person and online FGDs generated rich discussions, the in-person sessions allowed for more dynamic interaction and spontaneous exchanges. However, the online format enabled broader participation despite logistical constraints, and no substantial differences in thematic depth were observed across modes. The facilitator maintained a neutral stance during the discussions and was aware of his professional background as a pharmacist, which could potentially influence interpretations.

A semi-structured FGD guide was developed based on the study objectives, comprising open-ended and follow-up questions. The Socio-Ecological Model (SEM) was being used as theoretical framework that conceptualises individual, interpersonal, organisational, and policy-level determinants of health behaviour. This model informed the development of the FGD guide by structuring questions to explore determinants at four levels: individual (knowledge and practices), interpersonal (social norms and interactions), organizational (institutional roles and resources), and policy (regulatory frameworks). The guide explored participants’ perceptions of community practices, barriers, and facilitators related to unused medicine management across these levels. The interview questions were refined following expert consultation (SAK and CW) and pilot testing. In addition, a brief demographic questionnaire capturing participants’ roles, gender, institutional affiliation, and years of experience was administered prior to the discussion.

The guide covered two main domains: (1) stakeholders’ perspectives on pharmaceutical waste management in the community and (2) perceived challenges in implementing proper management practices. The topic guide was structured according to the four levels of the Socio-Ecological Model. Examples of guiding questions are presented in Table 1, while the full FGD guide, including additional probing questions, is provided in the Additional File.

**Table 1.** Example of FGD topic guide based on the socio-ecological model.

SEM level	Focus area	Example questions
Individual	Current practices, knowledge, and perceptions	*“Can you describe what people usually do with leftover or expired medicines?”*
Interpersonal	Social influences on medicine disposal practices	*“Based on your perspective, are there any factors in community such cultural, social, or economic that influence this behavior?”*
Organizational	Institutional roles and resources	*“How do health facilities or pharmacies manage unused medicines from patients?”*
Policy	Regulations and governance	*“What policies or programs that provide guidance for households to dispose their unused medicines?”*
Facilitators	Opportunities and enablers	*“What encourages or supports community efforts to improve unused medicine management?”*

### Data analysis

All data were transcribed verbatim into five separate Microsoft Word files, each corresponding to one FGD or interview session and containing both the guiding questions and participants’ responses. The principal investigator verified the accuracy of the transcripts by repeatedly listening to the recordings and cross-checking them against the written text. Transcripts were shared with participants for validation and feedback evaluation. Transcripts were then imported into NVivo version 12 for coding and analysis. A thematic analysis was applied using an inductive and reflexive approach due to its flexibility ( Jakobsen et al., 2023) and guided by Braun and Clarke’s six-phase framework. First, the research team familiarized itself with the data through repeated reading of the transcripts. Second, open coding was conducted to capture meaningful segments of text. Third, codes were organized into initial categories, which were subsequently refined into sub-themes. Fourth, emerging sub-themes were reviewed and synthesized into main themes that reflected shared concepts across the data. Fifth, these themes were critically examined and refined to ensure coherence with the dataset. Finally, each theme was clearly defined and labeled to capture its essence ( Braun & Clarke, 2006). Given the multi-stakeholder design, the study prioritized diversity of perspectives rather than saturation within a single group. Coding and theme development were discussed collaboratively among members of the research team to enhance analytical rigor, reduce individual bias, and ensure the dependability of the findings. Themes were subsequently organized using the Socio-Ecological approach to illustrate how barriers and facilitators operate across individual, community, organizational, and policy levels. This approach ensured that the analysis captured multi-level influences on unused medicine management ( Golden & Earp, 2012). In addition, this study followed the consolidated criteria for reporting qualitative research (COREQ) and its 32-item checklist to enhance transparency and trustworthiness in the reporting process ( Tong et al., 2007).

### Rigour and trustworthiness of the study

To ensure rigour and trustworthiness, this study followed Lincoln and Guba’s four evaluative criteria. Credibility was enhanced through triangulation across diverse stakeholder groups and peer debriefing among the research team. Dependability was ensured by maintaining a detailed audit trail of methodological decisions, coding processes, and theme development. Confirmability was strengthened through collaborative analysis and transparent documentation to minimize individual bias. Transferability was supported by providing a rich description of the study context, participants’ characteristics, and thematic findings, enabling readers to assess the applicability of results to other settings ( Lincoln & Guba, 1985).

## Results

### Characteristics of participants

Total number of participants were 43 with 2 participants withdrawing due to scheduling issue. No repeat interviews were conducted. Most participants were adult between 25-55 years (median = 41). The majority were female (34.8%), reflecting gender composition of community organization. Most had completed tertiary-level education, and several holding postgraduate degrees, particularly among policymakers, whereas within the PKK group showed more diverse educational background. Over half of the participants had worked for more than 10 years in their respective sectors, suggesting substantial institutional experience. Details of participants’ roles, gender, and work experience are presented in Additional File 1.

### Themes and subthemes

The research team led by RAMRS identified four interrelated themes. The foundational theme concerns community behaviour in managing unused medicines, including practices of storage and disposal, as well as the underlying reasons shaping these practices. Themes and subthemes used in this study are presented in Table 2.

**Table 2.** Themes, and sub-themes generated from the finding.

Theme	Sub theme
1. Community Practices in Managing Unused Medicines	• Storing medicines (including expired) • Disposal into household waste • Burning and burying medicines • Disposal into drainage systems • Use for non-medical purposes
2. Barriers to Proper Management	Individual level: • Limited knowledge and awareness • Attitudes and beliefs (e.g., “just-in-case”, perceived value) Interpersonal level: • Medicine sharing practices • Social norms and low prioritisation Organizational level: • Lack of disposal facilities • Limited human and financial resources • Absence of SOPs and service standards Policy level: • Regulatory gaps • Lack of budget allocation • Fragmented institutional roles
3. Facilitators for Proper Management	• Increasing public awareness (post-COVID-19) • Existing community and organizational programs • Availability of national guidelines • Opportunities for intersectoral collaboration

### Community behaviour in managing unused medicines

Participants described diverse behaviours related to unused medicines, including storing expired medicines at home, discarding them in household waste, burning or burying them, and even using them for non-scientific purposes. These practices illustrate how community members manage unused medicines in the absence of formal disposal facilities. Behavior and quotation are presented in Table 3.

**Table 3.** Improper community behavior in managing medicines.

Sub theme	Illustrative quotes
Storing medicines at home, even when expired	*”People often keep one or two tablets and stop using them once they feel recovered. Later, when officers visit, the medicines are neatly arranged but already covered in dust.”* (P36)
Disposing into trash	*”From the community’s side, there are some patients who behave like once there are no more symptoms, they store the medicines. Most of them get the medicines without prescriptions, especially antibiotics, and then they just throw them away into the trash.”* (P38)
Burying medicines in the ground	*“What I know is that expired medicines are first removed from their packaging. The packaging is thrown away, while the medicine itself is crushed. The final step is usually to dig a hole, bury the medicine, and cover it with soil.”* (P24)
Burning medicines	*“For the community here, medicines are still mixed with household waste. There is no waste collection system from the Environmental Agency like in the city. So, in this area, medicines are still burned together with household waste.”* (P14)
Pouring into drainage systems	*“For unused syrups, sometimes when they are no longer good, people simply pour them into the drainage system.”* (P22)
Disposing into fish ponds	*“The disposal pattern is either burning together with other household waste, or throwing them into the fish ponds located beside or behind their houses.”* (P6)
Using unused medicines for non-scientific purposes	*“I enjoy growing orchids. I take unused medicines and then put them into a spray bottle, and spray them on the plants when I see pests. Now the flowers look fresh and healthy. I believe the medicines help.”* (P30) *“In my family, there is a belief passed down through generations that adding paracetamol when boiling meat can make it more tender.”* (P25)

### Mapping of barriers and facilitators in improper medicine disposal using a socio-ecological approach

**Figure 1.**
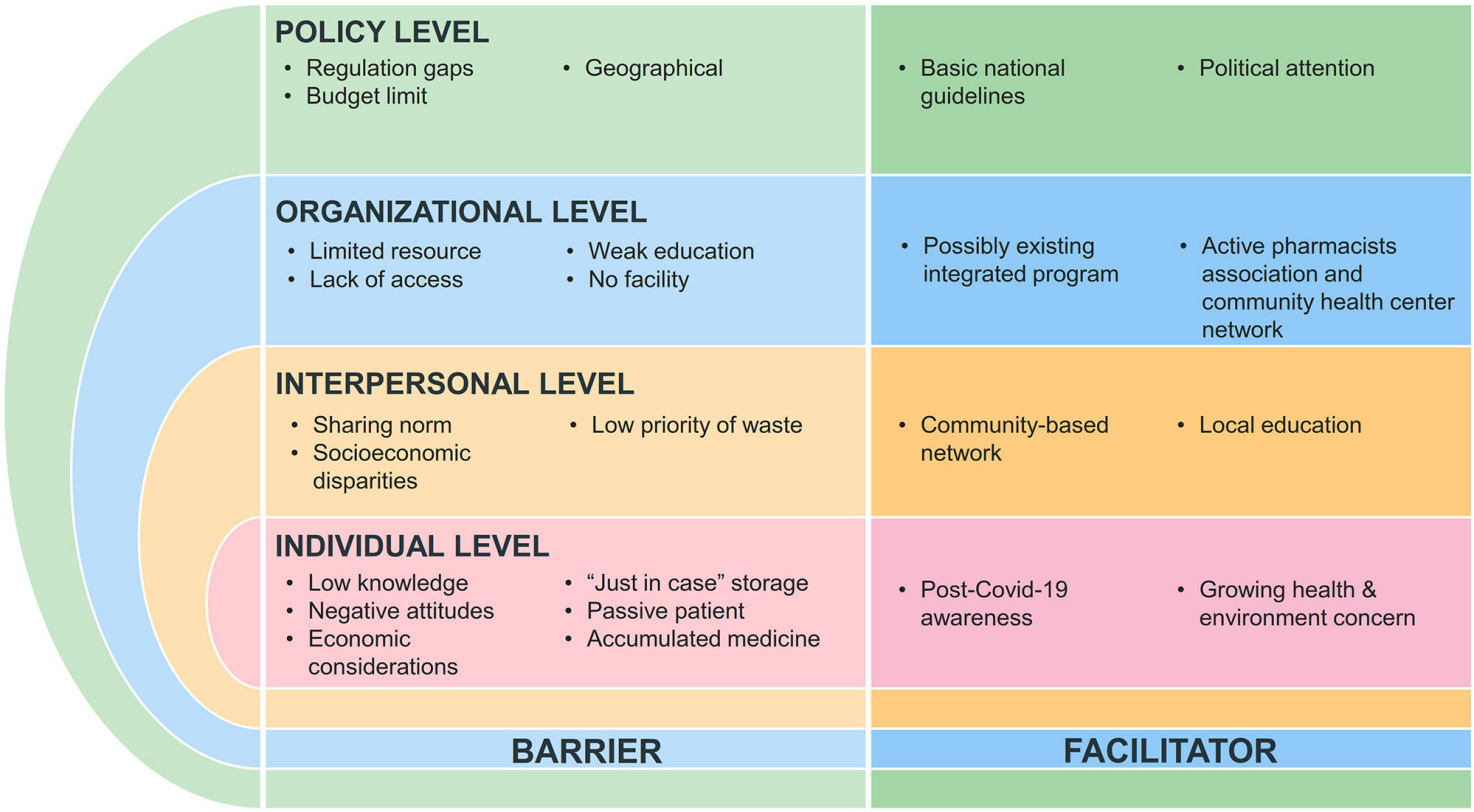
Barriers and facilitators across socio-ecological levels influencing leftover medicine management.

In addition to behavioural observations, our findings revealed multiple barriers and facilitators that influence community practices in managing unused medicines. To better illustrate the complexity of these interrelated factors, we organised them using the Socio-Ecological Model ( Golden & Earp, 2012), which distinguishes individual, interpersonal, organisational, and policy levels. As illustrated in Figure 1, these factors do not operate independently but interact to form a system of influence. For example, individual tendencies such as storing medicines for future use are reinforced by interpersonal practices, including medicine sharing within families and communities. At the same time, these behaviours are sustained by organisational limitations, such as the absence of disposal facilities, and further shaped by broader policy constraints, including unclear regulations and limited resource allocation. This interconnected structure highlights how barriers and facilitators are embedded across levels rather than occurring in isolation. A complete mapping of these factors can be found in Table 4 and Figure 1.

**Table 4.** Socio-ecological approach in mapping the barriers and facilitators of unused medicine disposal.

Level	Barrier	Facilitator
**Individual**	− Low knowledge in waste management − Negative attitudes toward leftover medicines − Economic considerations − “Just in case” practice − Passive patient − Accumulated medicine	− Increased community participation in waste management following COVID-19 − Stronger health and environmental awareness.
**Interpersonal**	− Sharing medicines norm − Socioeconomic disparities − Low prioritization of waste management	− Experience of community-based networks that could be mobilized for household monitoring and education.
**Organizational**	− Ease of accessibility − Lack of service standards − Institutional resource limitations − Ineffective education − Absence of facilities	− Existing institutional programs that could integrated with medicine waste issues. − Readiness of Pharmacist associations and health centers to collaborate in future initiatives.
**Policy**	− Regulatory limitations − Budgetary constraints − Institutional authority limitations − Geographical barriers	− Use of related national guidelines by local office. − Ministerial and political attention to the issue. − Growing intersectoral collaboration initiatives.

### Individual level

At the individual level, the main issue was affected by attitude and cultural beliefs. Many viewed discarding medicines as wasteful or financially disadvantageous, leading to the habit of storing them for “just in case” use. Passive patients also contribute to accumulated medicine in the home as they tend not to question any medication they received from physicians. We could state that all of these were caused by limited knowledge.

*“Sometimes people keep unused medicines, therefore they or their acquaintances can use them later without buying new ones. There is also a cultural belief that throwing medicines away is wasteful.”* (P41)

*“Unused medicines are more common among adults, especially the elderly, who tend to accept whatever their medication is and accumulate them without checking expiry dates.*” (P42)

Despite these challenges, some facilitators were identified at this level. Respondents observed a gradual increase in public awareness and participation following the COVID-19 pandemic, as communities became more health-conscious.

*“From our experience, people were willing to adopt proper waste management practices, especially after COVID-19 made them more health-conscious.”* (P8)

### Interpersonal level

At the interpersonal level, social norms and family practices played a significant role in shaping behaviours. The sharing of leftover medicines among relatives or neighbours was reported as a common practice, particularly in lower-income communities. They also viewed medicine disposal as minor issue and possibly didn’t want to contribute financially, as in current conditions, people often refused to pay the waste transportation fee. Participants from the Health Office once mentioned their experiences in finding Digoxin being sold in a small kiosk (warung) due to the seller’s ignorance.

*“People often give unused medicines to their family or friends if they have similar symptoms, rather than disposing of them.”* (P36)

### Organizational level

At the organizational level, several structural and institutional barriers emerged. Respondents consistently mentioned the absence of standardized procedures and limited disposal facilities. Health facilities also faced workforce and resource constraints that limited their ability to conduct consistent education and outreach. Mostly, participants from the Health Office and the Community Health Center stated their problem in not having enough resources for even standard pharmaceutical services.

*“Even when people are aware that medicines must be properly managed, they still ask, ‘Where should this be taken?’ because the facilities are limited.”* (P5)

### Policy level

At the policy level, barriers related to regulation, coordination, and budget allocation were dominant. Respondents emphasized the absence of clear national or local regulations governing household pharmaceutical waste, leading to uncertainty among implementers. Fragmented institutional responsibilities also hampered effective management, as no special taskforce was appointed to be in charge.

*“Currently, no mandatory regulations govern this issue. If such policies existed, they would come with programs and budgets.”* (P42)

Nevertheless, participants recognized several emerging facilitators at this level, including the existence of national guidelines and the growing political attention, such as from the Coordinating Ministry for Human Development and Cultural Affairs or the Regent, toward pharmaceutical waste.

*“The Directorate General of Pharmaceutical Services issued Decree No. HK 02031708/2021, which provides household-level guidelines for managing expired and damaged medicines.”* (P8)

Intersectoral initiatives and community-based programs such as Climate Village (Kampung Iklim), or Eco-friendly Village (Kangraling) by the Environmental Agency, and Pharmacists Go to Village (Sonagar Mapay ka Lembur) by the Pharmacists Association were viewed as potential platforms to integrate unused medicine management into broader environmental and health policies.

## Discussion

This study explored how stakeholders from different institutions view unused medicines as an issue in the community. We identified their perspectives on community practices, barriers, and facilitator related to unused medicines management in Garut regency. Currently, pharmaceutical waste management in Indonesia operates without a formalized system ( Subadi, 2022). Our findings mentioning that households rely on informal disposal methods, while health facilities lack standardized protocols and designated return mechanisms. Responsibilities are fragmented across institutions, resulting in minimal coordination and limited service provision. By using a socio-ecological approach as an analytical framework, in this context, we can explain that community behaviours appear to be shaped by structural constraints rather than solely individual decision-making, organizational, and policy level.

### Community behaviours and household practices

Although these practices pose potential environmental and health risks, their persistence reflects not only limited awareness but also the absence of accessible and acceptable disposal options. In this context, communities tend to rely on disposal methods that are readily available and practical in everyday life. Some medicines are also repurposed for non-medical uses, such as tenderizing meat or treating plants, reflecting perceptions of medicines as valuable and versatile resources rather than strictly regulated health commodities. These practices illustrate adaptive behaviours shaped by local knowledge, beliefs, and resource constraints.

These practices pose potential environmental and health risks. For instance, low-temperature burning of medicines may release toxic air pollutants and heavy metal residues ( Alnahas et al., 2020), while pharmaceutical compounds have been detected in landfill leachate (e.g., multiple active ingredients identified in China). In addition, the repurposing of medicines for non-medical uses such as food preparation or plant treatment raises concerns regarding toxicity and unintended human exposure pathways, including potential uptake of pharmaceutical compounds by plants ( Ezugwu et al., 2023; Tanoue et al., 2012). However, the persistence of these practices reflects not merely a lack of awareness but limited access to appropriate disposal options and the absence of clear guidance, particularly in communities with constrained resources and information.

Similar patterns have been observed in both low and middle income countries, where unsafe disposal practices are often linked to limited infrastructure and weak regulatory systems ( Mohammed & Al-Hamadani, 2023; Mostafanejad et al., 2025), as well as in high-income settings where formal return programs exist but are underutilized ( Kelly et al., 2018). Taken together, these findings suggest that unsafe disposal practices are not solely driven by individual-level factors but are embedded within broader systemic conditions. Without clear guidance, accessible facilities, and institutional support, communities are more likely to adopt informal practices that are practical, even if potentially unsafe.

### Individual-level barriers to the proper disposal of unused medicine

Barriers at the individual level reflect an interplay between knowledge, attitudes, and contextual realities. Limited understanding of waste management and pharmaceutical risks has been widely reported to reduce the likelihood of safe practices ( Ayele & Mamu, 2018; Kusuma et al., 2023; Sim et al., 2018). In this study, community representatives acknowledged gaps in knowledge, while health and environmental stakeholders emphasised challenges in basic waste practices, such as sorting household waste. Evidence suggests that engagement in waste management behaviours is often driven by perceived environmental risk, which remains low in many settings ( Cai et al., 2024).

However, these behaviours cannot be explained by knowledge deficits alone. Medicines are frequently perceived as valuable commodities, leading households to retain them for future use. As illustrated by participants, economic considerations and cultural beliefs, such as avoiding “wastefulness” encourage ‘just-in-case’ storage and reuse. Previous studies have similarly shown that medicine reuse may be perceived as cost-saving, particularly for more expensive treatments ( Alhamad et al., 2018). In this context, retaining medicines is not necessarily irrational, but reflects adaptive decision-making under financial and access constraints.

Accumulated medicine could be directly caused by expired products, medicines left behind after patient death, and drugs dispensed in loose packaging without clear labeling. Demised patient potentially left half of their medication unused and could be end in their family not using it anymore ( Ekedahl, 2006). Loose packaging led to inadequate label or unclear instruction and could end with patient confusion and tend to not using the medicine. These phenomenon observed in Srilanka which inadequate label and packaging mostly happened to medicine obtained from public sector compared to private sector ( Athuraliya et al., 2016). Passive patient attitudes, particularly among older adults, contributed to stockpiling behavior, whereas more informed patients tend to manage medicines more appropriately after discharge ( McTier et al., 2015). These findings suggest that individual-level barriers are not solely the result of insufficient knowledge or negative attitudes, but are closely linked to broader economic, cultural, and health system conditions. This highlights the importance of moving beyond education-focused interventions towards more comprehensive strategies, including rational prescribing, improved dispensing practices, and clearer patient guidance.

### Interpersonal level barriers to the proper disposal of unused medicine

Our findings emphasized that social aspect, like norms and socioeconomic disparities, shape the management of unused medicines within households and communities. The practice of sharing medicines was frequently reported, highlighting cultural norms where medicines are perceived as communal, valuable resources rather than personal prescriptions. This practice could be seen as a positive thing, and participant often have stated that this was an act of justice in terms of saving cost and improving their social relationship ( Beyene et al., 2016). However, it raises some concern regarding increasing risk of drug resistance ( Dimitrov et al., 2012) or kidney failure ( Makówka et al., 2015) in the population. Similar patterns have been documented in both Asian ( Song et al., 2022) and African contexts ( Obol et al., 2018), where medicine reuse and sharing are normalized. These findings suggest that medicine sharing is not simply a result of poor knowledge or irrational behaviour, but rather a rational response to economic considerations and limited access to healthcare resources. As reflected in participants’ views, discarding medicines is often perceived as wasteful (P41), indicating that such practices are shaped by practical decision-making within constrained environments.

Furthermore, participants from higher economic status mentioned that some of their worker often asked for their leftover medicines to be taken home. This socioeconomic discrepancies explained as lower-income groups that rely on leftover medicines from others to reduce healthcare costs as also seen in methadone maintenance patient ( Caviness et al., 2013). Finally, proper disposal of unused medicines is rarely prioritized, as it is perceived as a burden without immediate benefits to households. Some participant mentioned that this act of proper disposal didn’t benefit them, difficult to implement or just have no motivation in doing therefore. The lack of tangible incentives, coupled with limited awareness of environmental and health risks, leads to low community engagement in safe disposal practices. Study in middle income country also mentioned incorrect practice of disposal when no incentives provided ( Lago et al., 2022). Together, these barriers illustrate how economic constraints and cultural norms strengthen improper practices at the community level, highlighting the need for both educational interventions and system-level solutions to provide more accessible and acceptable disposal pathways. Importantly, these interpersonal dynamics do not operate in isolation but are reinforced by broader structural conditions. In the absence of accessible disposal systems and clear institutional guidance, sharing and storing medicines become practical alternatives for communities. This indicates that interpersonal norms both shape and are shaped by organizational and policy-level limitations, further sustaining unsafe disposal practices.

### Organizational-level barriers to the proper disposal of unused medicine

At the organizational level, barriers were primarily related to limitations in system capacity and service delivery, rather than a lack of stakeholder willingness. Pharmacists and the Health Office representatives consistently highlighted that medicines remain highly accessible in the community, both through informal outlets and prescribing practices within health facilities. Studies in similar settings have shown widespread availability of antibiotics in small retail outlets, often without prescription and sometimes in substandard packaging ( Hadi et al., 2010), driven by a combination of weak regulatory enforcement, market demand, and commercial incentives ( Belachew et al., 2021). While healthcare providers themselves may demonstrate adequate knowledge and attitudes toward appropriate medicine use ( Amin et al., 2022), these structural conditions contribute to an oversupply of medicines at the household level.

At the same time, stakeholders reported significant uncertainty in managing unused medicines due to the absence of clear standard operating procedures and limited institutional guidance. Existing regulations tend to focus on healthcare-generated waste, leaving household pharmaceutical waste insufficiently addressed ( Alfian, Azzahra, et al., 2024a), and awareness of available guidelines remains low, with little formal training provided ( Alfian, Rendrayani, et al., 2024b). This lack of clarity is compounded by the absence of accessible return systems, meaning that even when communities are willing to dispose of medicines properly, there are few practical options available. Evidence from other settings shows that the presence of medicine take-back facilities is strongly associated with safer disposal practices ( Ehrhart et al., 2020).

Institutional capacity further constrains implementation. Limited numbers of health workers and pharmacists often require prioritisation of clinical services, leaving little room for sustained education and monitoring of pharmaceutical waste. Although educational initiatives such as “Counseling, Information and Education” (KIE), “Let’s Throw Medication Waste” (ABSO), and “Smart Medicine Use Movement for Community” (Gema Cermat) aim to improve public awareness, their reach and effectiveness at the local level remain uneven ( Kristina et al., 2021; Wahyudi & Kristina, 2022). In aggregate, these findings suggest that organizational barriers are not simply operational challenges, but reflect broader system limitations in regulation, infrastructure, and resource allocation. As a result, even well-informed and motivated stakeholders face constraints in translating knowledge into practice. This reinforces the idea that unsafe medicine management is shaped by structural conditions, highlighting the need for coordinated institutional strengthening alongside community-level interventions.

### Policy-level barriers to the proper disposal of unused medicine

At the policy level, barriers reflect fundamental gaps in governance, coordination, and resource allocation that shape the overall system. Policymakers and regulatory stakeholders highlighted the absence of comprehensive regulations specifically addressing household pharmaceutical waste, resulting in unclear mandates and limited accountability across institutions. Similar gaps have been reported across ASEAN countries, where responsibilities for management remain fragmented and poorly defined ( Sapkota & Pariatamby, 2025; Ravinetto et al., 2025).

Financial constraints further limit implementation. The absence of dedicated funding at national and local levels restricts the ability of authorities to develop and sustain disposal programs. In contrast, evidence from OECD countries demonstrates that pharmaceutical waste systems are often supported through structured financing mechanisms, including government funding and extended producer responsibility schemes ( Organisation for Economic Co-operation and Development, 2022). This could raise the option of opening up into some scheme for managing pharmaceutical waste.

In addition, institutional responsibilities are distributed across multiple actors—including the Health Office, Environmental Agency, and professional associations—without clear coordination mechanisms. Stakeholders reported that this fragmentation leads to overlapping roles, limited authority, and insufficient human resources, constraining effective implementation. While collaborative approaches, such as community-based waste initiatives or multi-sector governance models, have shown potential in other contexts ( Syafriani et al., 2021; Song et al., 2025), their effectiveness depends on clear policy direction and institutional alignment.

Geographical factors further complicate implementation. Garut’s wide and diverse terrain limits consistent outreach and access to services, particularly in rural areas, where disparities in sanitation and healthcare infrastructure persist ( Irianti & Prasetyoputra, 2021; Ismail & Setyawan, 2025). Collectively, these findings suggest that policy-level barriers extend beyond regulatory gaps to cover broader structural limitations in governance, financing, and coordination. These constraints cascade downward, weakening organizational capacity and reinforcing unsafe practices at the community level. This underscores the need for integrated policy frameworks that align institutional roles, secure sustainable funding, and support equitable service delivery across regions.

### Facilitators and opportunities for intervention

Despite these challenges, several facilitators identified by participants indicate clear opportunities for intervention across multiple levels. Increased public awareness, particularly following the COVID-19 pandemic, suggests a growing receptiveness to health and environmental messages. This shift may reflect broader changes in risk perception and daily exposure to medical products, which have been shown to enhance collective awareness of healthcare-related waste ( Raesi et al., 2024; Alomari et al., 2021). At the policy and organizational levels, the existence of national guidelines provides a foundational framework that can be further operationalised at the local level. However, participants noted that these guidelines remain underutilised, highlighting the need for clearer implementation strategies and dissemination efforts.

Importantly, existing community and institutional platforms offer practical entry points for intervention. Programs led by PKK volunteers, environmental initiatives such as Kampung Iklim (Climate Village), and pharmacist-led campaigns by professional organizations demonstrate the potential for integrating unused medicine management into ongoing activities. These platforms could be leveraged to support community-based collection systems, education, and monitoring, particularly if supported by formal coordination and resources. Experiences from other settings further illustrate the potential of multi-stakeholder approaches. For example, medicine take-back initiatives involving coordinated roles across healthcare providers, regulators, environmental agencies, and community organisations have been successfully implemented ( Abahussain et al., 2024). Adapting such models to the local context could involve mobilising PKK networks for household-level engagement, pharmacists for safe handling and collection, and local authorities for coordination and oversight.

Taken together, these facilitators highlight that opportunities for improvement already exist within the system but remain underutilised. Strengthening these elements through coordinated, multi-level strategies could enable more effective and sustainable management of unused medicines.

### Positioning findings within a socio-ecological and stakeholder perspective

Although the themes were derived inductively, their interconnections can be mapped onto different levels of influence, consistent with the socio-ecological model (SEM). The findings further indicate that each level is perceived differently by distinct stakeholder groups, reflecting how responsibilities and influence are distributed across the system. SEM conceptualises behaviour as shaped by interacting intrapersonal, interpersonal, organisational, community, and policy contexts, enabling the identification of both proximal and distal determinants and supporting the development of integrated, context-sensitive interventions, particularly in low- and middle-income settings ( Olaniyan et al., 2021; Salihu et al., 2015).

At the individual and interpersonal levels, PKK representatives and frontline pharmacists reported that medicine storage and sharing are driven by economic considerations, cultural beliefs, and limited awareness. These behaviours are closely linked to individuals’ knowledge, attitudes, and risk perceptions, often shaped by prior experiences and sociocultural norms ( Litchfield et al., 2021), and are reinforced within social networks such as family and neighbours. However, these practices are further shaped and constrained by organizational conditions. Pharmacists and Health Office representatives highlighted structural limitations, including the absence of standard operating procedures, inadequate disposal infrastructure, and limited human and financial resources, which restrict the translation of awareness into safe practices.

At the policy level, policymakers and regulatory actors identified gaps in formal regulations, unclear institutional mandates, and a lack of dedicated funding as key structural barriers. These constraints cascade downward, weakening organizational capacity and reinforcing individual-level behaviours, indicating that unsafe medicine management reflects systemic limitations rather than solely individual choices.

By applying SEM in this way, the findings highlight critical leverage points for intervention. Efforts focused solely on community education are unlikely to be effective without parallel investments in organizational infrastructure (e.g., accessible collection systems) and policy support (e.g., clear regulations and funding mechanisms) ( Davidson et al., 2018). The model also clarifies the complementary roles of stakeholders: community organizations such as PKK can influence household practices, pharmacists can facilitate safe handling and collection, and policymakers are essential in enabling these actions through regulatory and institutional support.

Therefore, addressing unused medicine management in Garut requires a coordinated, multi-level strategy that aligns these stakeholder roles. Interventions should move beyond targeting individual behaviour in isolation and instead prioritise system-level integration, where policy commitment, institutional capacity, and community engagement are strengthened simultaneously. Overall, the socio-ecological model provides a valuable framework for understanding how behaviour is shaped by dynamically interacting factors across multiple levels that continuously influence one another within specific contextual conditions.

## Conclusion

This study demonstrates that the management of unused medicines in Garut Regency is shaped by interconnected factors across individual, interpersonal, organizational, and policy levels. Stakeholders identified unsafe household practices, cultural norms of medicine reuse, limited institutional capacity, and fragmented regulatory responsibilities as key barriers. At the same time, increasing public awareness, existing national guidelines, and community-based initiatives provide important entry points for intervention. These findings suggest that improving pharmaceutical waste management requires moving beyond education-focused approaches towards coordinated, system-level strategies. One practical approach would be to pilot a community-based medicine take-back program by leveraging existing structures. For example, PKK volunteers could be trained to identify and collect unused medicines during routine household visits, with safe handling and temporary storage supported by local Puskesmas pharmacists. These medicines could then be periodically collected and managed by the Health Office in coordination with regulatory bodies such as BBPOM, supported by clear guidelines and designated funding. In parallel, strengthening organizational capacity through the development of standard operating procedures and integrating medicine disposal into existing health promotion programs could enhance sustainability. At the policy level, clearer regulatory mandates and dedicated budget allocation are needed to formalize roles and ensure continuity of such initiatives. Overall, aligning community networks, healthcare providers, and policymakers within a coordinated multi-level framework offers a feasible and context-sensitive pathway to improve the safe management of unused medicines in Garut and similar settings.

### Strength and limitation

A key strength of this study lies in the inclusion of diverse stakeholder groups, which enabled a multi-level understanding of unused medicine management from community, professional, and policy perspectives. The qualitative design, combined with separate focus group discussions, allowed for the exploration of context-specific barriers and facilitators that may not be captured through survey-based approaches. However, several limitations should be considered. First, this study was conducted in a single regency in West Java, and the findings may not be fully transferable to other regions of Indonesia with different sociocultural contexts, geographic characteristics, and healthcare infrastructures, such as those in Eastern Indonesia. These contextual differences may influence both access to services and community practices related to medicine use and disposal. Second, as the study primarily involved institutional stakeholders, the findings may reflect organisational and professional perspectives more strongly than those of the general community. While these stakeholders interact closely with the public, their views may not fully capture the lived experiences and decision-making processes at the household level. Finally, although the socio-ecological model provided a useful framework for organising the findings, it may simplify the complex and dynamic interactions between levels of influence. In particular, the model does not capture the relative weight or power of different factors, nor how these may shift across contexts, which may limit its ability to fully explain causal relationships.

## Ethical considerations

Ethical approval for this study was obtained from the Ethics Committee of the Faculty of Medicine, Public Health, and Nursing, Universitas Gadjah Mada (Number KE/FK/0965/EC/2025). All participants provided written informed consent prior to data collection. Anonymity and confidentiality were ensured by removing all personal identifiers and reporting data only at the institutional or role level.

## Data Availability

Figshare: Underlying data for Views, Barriers and Facilitators of Policymakers, Pharmacists and Health Community Representative in Managing Unused Medicine in a Socioeconomically Diverse District in Indonesia. https://doi.org/10.6084/m9.figshare.31015444 ( Syamsudin et al., 2026) This project contains participant demographic characteristics. Access to the interview transcripts is restricted due to ethical and confidentiality considerations. Researchers may request access by contacting the author at aldizal@uniga.ac.id Data are available under the terms of the Creative Commons Attribution 4.0 International license (CC BY 4.0). Figshare: Extended data for Views, Barriers and Facilitators of Policymakers, Pharmacists and Health Community Representative in Managing Unused Medicine in a Socioeconomically Diverse District in Indonesia. https://doi.org/10.6084/m9.figshare.31015444 ( Syamsudin et al., 2026). This project contains the following extended data:
1. FGD Guide (Guide used to facilitate focus group discussions and interviews). 2. Thematic coding framework (Example of the coding framework developed during qualitative analysis). 3. Informed consent template (Template of the informed consent form provided to participants). Data are available under the terms of the Creative Commons Attribution 4.0 International license (CC BY 4.0). Repository: COREQ checklist and for ‘Views, Barriers and Facilitators of Policymakers, Pharmacists and Health Community Representative in Managing Unused Medicine in a Socioeconomically Diverse District in Indonesia’. https://doi.org/10.6084/m9.figshare.31015321.
