# Supplementary material for: Views, Barriers and Facilitators  of Policymakers, Pharmacists and Community Health Representative in Managing Unused Medicine in a Socioeconomically Diverse District in Indonesia
Source: F1000Res. 2026 May 4;15:314. Originally published 2026 Feb 24. [Version 2] doi: 10.12688/f1000research.177671.2 (PMC13156558; doi:10.12688/f1000research.177671.2)
Supplement: Peer review history

# Reviewer report: Reviewer response for version 2

**DOI:** 10.5256/f1000research.198938.r481405

**Published:** 2026-05-08

**Authors:** Liz Breen

**Affiliations:** 1 University of Bradford, Bradford, England, UK

The authors have completed the revisions to the expected standard and the paper is a more thorough version. Well done.

Is the work clearly and accurately presented and does it cite the current literature?

Partly

If applicable, is the statistical analysis and its interpretation appropriate?

Not applicable

Are all the source data underlying the results available to ensure full reproducibility?

Partly

Is the study design appropriate and is the work technically sound?

Yes

Are the conclusions drawn adequately supported by the results?

Partly

Are sufficient details of methods and analysis provided to allow replication by others?

Yes

Reviewer Expertise:

Pharmaceutical supply chain, design, improvement and sustainability.

I confirm that I have read this submission and believe that I have an appropriate level of expertise to confirm that it is of an acceptable scientific standard.

# Reviewer report: Reviewer response for version 2

**DOI:** 10.5256/f1000research.198938.r481406

**Published:** 2026-05-06

**Authors:** Danladi Chiroma Husaini

**Affiliations:** 1 University of Belize, Belmopan, Belize

The authors have addressed all my concerns. I have no further comments. I wish them success.

Is the work clearly and accurately presented and does it cite the current literature?

Yes

If applicable, is the statistical analysis and its interpretation appropriate?

Partly

Are all the source data underlying the results available to ensure full reproducibility?

Yes

Is the study design appropriate and is the work technically sound?

Yes

Are the conclusions drawn adequately supported by the results?

Yes

Are sufficient details of methods and analysis provided to allow replication by others?

Yes

Reviewer Expertise:

Experiential learning, Ethnopharmacology, Toxicology, Pharmacy education, Therapeutics, Climate change and health, Public Health, Environmental Health.

I confirm that I have read this submission and believe that I have an appropriate level of expertise to confirm that it is of an acceptable scientific standard.

# Reviewer report: Reviewer response for version 1

**DOI:** 10.5256/f1000research.195928.r462273

**Published:** 2026-03-18

**Authors:** Liz Breen

**Affiliations:** 1 University of Bradford, Bradford, England, UK

Really interesting focus for investigation. I would opt to bring in your socio-ecological approach earlier as it underpins your study, and the content of your introduction works within this realm. 

 Can we have some additional insight into what the government position is on pharmaceutical sustainability generally even if this does not drill down to household waste. There has to be a clear indication why this research is needed at this time and how it will impact. 

 In your introduction (last paragraph) you state the following - can you deliver to this given you have only examined on location? I would advocate that there could be learning for other LMICs but it isn't a given as it will require interpretation. 

 ......... and provides evidence to inform the development of comprehensive strategies for pharmaceutical waste management in LMIC settings.

 Methods - This section is repeated:

 Eligible participants were representatives from selected institutions who possessed broad knowledge of medicine management, provided pharmaceutical services, and/or had experience in community health. They were required to have at least one year of professional experience in a related field and to obtain an official recommendation from their institutional leader. Participants were excluded if they had less than one year of relevant professional experience, did not receive formal approval from their institution, were on temporary leave or not actively engaged in their professional duties during data collection, declined to provide informed consent, or had potential conflicts of interest that might compromise the integrity of the study. No prior relationship was established between the researcher and participants before the study commenced.

 I would advocate a separate and more comprehensive section on Ethics. Data collection should have its own identity.

 Table 1 insights are very limited, can we see the additional line of questioning?

 34,8% should be recorded as 34.8%

 Fig 1 are awareness post Covid etc facilitators or is it the action taken because of these that are facilitators? On their own they are inanimate and don't have an impact. 

 Individual level - why do you say could be? Surely the data tells you it is or isn't as this is speculative. - at the individual level, the main issue could be affected by attitude and cultural beliefs.

 Where you have a respondent, you should put in their role.

 Given this is a qualitative piece of work with focus group discussions the results are very quickly reduced to tables etc. Whilst the presentation in this form is help and is an excellent synthesis of data, I would expect to see it supplemented by rich data in the form of more quotes, even if these are tabularised. 

 Discussion - In the discussion it would be helpful to map the status quo with the stakeholders, what current waste management practice looks like. Do you have this information?

 Your paper would benefit from a grammar check e.g.  Recent study in China told us .......would read better as A recent study set in China advised that..... 

 from waste landfill slide) - why is this ) here?

 Can the findings as discussed here be narrowed down to a group e.g. which stakeholder group was burning medicines? Or putting them into landfill to correlate with the China example? 

 It's difficult to know what content in the Discussion at times the study findings v is previous literature/research. This should be clear. 

 Referencing accuracy - remove W (W. Song et al., 2025).

 Why are the barriers broken down by the different levels but not the facilitators - because of the volume reported? 

 There is interesting data here but the depth of analysis in the discussion can be enhanced with insight into discrete stakeholder views - currently they are aggregated so difficult to know which should be targeted for action, which could have the most influence in expediting change etc. 

 The socio-ecological model could be introduced in more depth to determine its influence on this study. This would be applicable to both the introduction and the Discussion. I encourage the authors to explore this section further and insert the additional content to strengthen this paper and its offer. 

 Good luck with your revisions.

Is the work clearly and accurately presented and does it cite the current literature?

Partly

If applicable, is the statistical analysis and its interpretation appropriate?

Not applicable

Are all the source data underlying the results available to ensure full reproducibility?

Partly

Is the study design appropriate and is the work technically sound?

Yes

Are the conclusions drawn adequately supported by the results?

Partly

Are sufficient details of methods and analysis provided to allow replication by others?

Yes

Reviewer Expertise:

Pharmaceutical supply chain, design, improvement and sustainability.

I confirm that I have read this submission and believe that I have an appropriate level of expertise to confirm that it is of an acceptable scientific standard, however I have significant reservations, as outlined above.

# Response

**Published:** 2026-04-16

**Authors:** Aldizal Mahendra

Dear Dr Liz Breen

 Thank you for your insightful and constructive feedback. We have carefully revised the manuscript accordingly:

1. SEM introduced earlier: The socio-ecological model has now been introduced earlier in the Introduction and further elaborated to clarify its role in framing the study.
2. Government position on pharmaceutical sustainability: Additional context on national policy and pharmaceutical waste management frameworks has been incorporated in the Introduction
3. Claim on LMIC relevance: We refined the statement to avoid overgeneralisation and clarified that findings may offer transferable insights with contextual interpretation.
4. Repeated Methods text: The duplicated participant eligibility section has been removed.
5. Ethics section: A separate and more detailed Ethics section has been added.
6. Data collection clarity: Data collection procedures have been expanded and separated clearly from recruitment.
7. Table 1 depth: Additional explanation of the FGD guide and its SEM basis has been included.
8. Figure 1 clarification: Figure 1 is now better integrated into the Results section and explicitly explained.
9. Speculative language: Revised to reflect evidence-based statements.
10. Use of participant roles: Participant roles are now consistently indicated in the findings.
11. Limited quotes: Additional quotes have been incorporated to enrich qualitative depth.
12. Discussion clarity: Revised to clearly distinguish findings from supporting literature.
13. Grammar and formatting issues: The manuscript has undergone thorough language editing and correction.
14. Stakeholder-specific insights: The discussion now better differentiates perspectives across stakeholder groups.

We hope that these revisions have addressed the your concerns and have strengthened the clarity, rigor, and overall contribution of the manuscript. We sincerely appreciate the reviewer’s insightful comments, which have been invaluable in improving the quality of this work.

# Reviewer report: Reviewer response for version 1

**DOI:** 10.5256/f1000research.195928.r464363

**Published:** 2026-03-12

**Authors:** Danladi Chiroma Husaini

**Affiliations:** 1 University of Belize, Belmopan, Belize

**Dear Authors,**

 First and foremost, I would like to commend you on tackling a critically important and under-researched public health and environmental issue. The problem of unused medicines is a global concern, and your focus on Indonesia, a country with a complex socio-economic landscape, is both timely and necessary. The decision to move beyond patient-level analysis and engage with a diverse group of stakeholders—policymakers, pharmacists, and community representatives—is a significant strength of your work. By applying the Socio-Ecological Model (SEM), you have laid a strong foundation for understanding the multi-layered challenges and opportunities in managing pharmaceutical waste. This is a valuable contribution that has the potential to inform real-world policy and practice. The following feedback is intended to help you refine and strengthen your manuscript for indexing.

 General Assessment

 This is a well-conceived qualitative study with a robust methodology. The use of focus group discussions (FGDs) and thematic analysis is appropriate for your research aims. The findings are insightful and clearly demonstrate the interplay between individual behaviors, community norms, institutional constraints, and policy gaps. The manuscript is well-structured, but there are several areas where clarity, depth, and presentation can be significantly improved to enhance its impact and meet the standards of a high-quality journal.

 Major Recommendations for Revision

 These are the key areas that require your attention to strengthen the manuscript's core arguments and contribution.

**Sharpen the Focus and Introduction:**

1. **Clarify the Research Gap:** The introduction does a good job of outlining the problem. However, the specific research gap you are filling could be stated more powerfully. You mention that existing research focuses on individual patients or pharmacist *willingness*, but you don't explicitly state that no one has yet brought these three stakeholder groups (policymakers, pharmacists, community reps) together in a single, comparative qualitative study in the Indonesian context. Explicitly stating this as your unique contribution will make your paper's significance immediately clear from the outset.
2. **Streamline the Argument:** The introduction could be more concise. For instance, the paragraph beginning "In high-income countries..." (Page 3) could be merged with the following one about Indonesia to create a sharper contrast between established systems and the local gap.

**Strengthen the Methods Section:**

1. **Consistency is Key:** There is a significant redundancy in the "Participant sampling and recruitment" section (Page 4). A large block of text describing eligibility criteria is repeated verbatim. Please remove the duplicate text to ensure a clean and professional presentation.
2. **Provide More Detail on the FGDs:** You mention that two FGDs were in-person and two were via Zoom. It would be valuable to briefly comment on whether you observed any differences in the dynamics or richness of data between these two modes. Did the online setting affect participation or the depth of discussion? Acknowledging this adds transparency.
3. **Clarify the Role of the SEM:** You state the FGD guide was "based on the Socio-Ecological Model." However, the guide questions presented in Table 1 are thematically organized. It would be helpful to briefly explain *how* the SEM informed the guide's development. For example, did you deliberately craft questions to probe each of the four SEM levels?
4. **Enhance the Presentation of Results:**

1. **Refine Table 2:** Table 2 (Themes and sub-themes) currently mixes themes and raw findings. For example, "Low community knowledge in waste management" is a finding, not a sub-theme. "Reasons for having unused medications" is too broad. I recommend restructuring this table to reflect your analytical process better:

- **Theme 1: Community Practices in Managing Unused Medicines** (Sub-themes: Storing medicines; Disposal into garbage; Burning; Disposal into water systems; Alternative uses).
- **Theme 2: Barriers to Proper Management** (Sub-themes: Individual-level barriers [knowledge, attitudes, beliefs]; Interpersonal-level barriers [medicine sharing, social norms]; Organizational-level barriers [lack of facilities, resources]; Policy-level barriers [regulatory gaps, budget]).
- **Theme 3: Facilitators for Proper Management** (Sub-themes: Growing public awareness; Existing organizational programs; National policy guidelines; Intersectoral collaboration). This structure would make your findings much clearer and directly map onto your SEM analysis.

 

- **Integrate Figure 1 More Deeply:** Figure 1 is a fantastic visual summary of your findings. However, it is introduced almost as an afterthought. In the text, you should explicitly guide the reader through the figure. For example: "As illustrated in Figure 1, the barriers are not isolated but form a system of influences. For instance, the individual-level barrier of 'economic considerations' (storing for 'just in case') is reinforced by the interpersonal norm of 'medicine sharing' and the organizational-level 'absence of facilities,' all within a policy environment of 'regulatory limitations.'"

 

**Deepen the Discussion and Conclusion:**

1. **Elevate the Discussion Beyond Description:** The discussion currently does an excellent job of reiterating your findings and linking them to the literature. To make it more impactful, try to synthesize your findings into a higher-level argument. For example, you could argue that "The persistence of unsafe practices is not a failure of individual rationality, but a rational response to systemic failures." This reframes the problem from one of patient education to one of health system and policy design. Use your data (e.g., P41's quote about wastefulness) to support this.
2. **Make the Recommendations Actionable:** In your conclusion, you state the need for "comprehensive and coordinated strategy." This is a great starting point. Now, based on your findings, can you offer one or two concrete, actionable recommendations for Garut or similar districts? For example: "Based on the facilitator of existing community networks, a pilot program could be designed that trains PKK volunteers to collect unused medicines during their household visits, which are then collected by pharmacists from the local Puskesmas, with clear guidance and support from the Health Office and BBPOM." This would demonstrate the practical value of your research.
3. **Acknowledge Limitations More Specifically:** The limitations section is good, but could be more specific. Instead of "may limit generalizability," you could say, "As a single-regency study in West Java, the findings may not be transferable to regions with different cultural norms, geographic characteristics (e.g., island communities), or healthcare infrastructures, such as Eastern Indonesia." This shows a deeper reflection on your study's context.

Minor Recommendations for Revision

 These are minor points that will improve the manuscript's clarity and professionalism.

- **Copyediting:** There are several typographical and grammatical errors throughout the manuscript. For example:

1. Page 1: "Yogakarta" should be "Yogyakarta."
2. Page 2: "ConclusionConclusion" is a duplicate.
3. Page 12: "Positioning findings within a socio- ecological perspective" is repeated as a subheading.
4. Page 12: "Facilitators and opportunities for intervention" is repeated as a subheading.
  A thorough proofread by a native English speaker or a professional editing service is highly recommended.

 

- **Terminology:** The term "health community representative" is a bit unclear. Using "community health representatives" or "Family Welfare and Empowerment Team (PKK) representatives" throughout would be more precise.

 

- **Figures and Tables:**
  1. **Table 3:** The quote from P30 about using medicines for orchids is fascinating. It powerfully illustrates the theme of "non-scientific purposes." Its value might be slightly diminished by the less clear quote from P25. Consider keeping P30 and finding a clearer quote to illustrate the meat-tenderizing myth, or simply describing this finding in the text without a direct quote.
  2. **Figure 1:** The resolution of the figure seems low in the PDF. Please ensure a high-resolution version is submitted.

As indicated earlier, this is a valuable piece of research that deserves indexing. The strength of your multi-stakeholder approach and the application of the Socio-Ecological Model provide a nuanced and comprehensive understanding of the issue. By addressing the recommendations above—particularly sharpening your unique contribution, refining the presentation of your themes, and deepening the discussion to offer more actionable insights—you will significantly enhance the manuscript's clarity, impact, and suitability for indexing. I look forward to seeing the final version of your important work.

Is the work clearly and accurately presented and does it cite the current literature?

Yes

If applicable, is the statistical analysis and its interpretation appropriate?

Partly

Are all the source data underlying the results available to ensure full reproducibility?

Yes

Is the study design appropriate and is the work technically sound?

Yes

Are the conclusions drawn adequately supported by the results?

Yes

Are sufficient details of methods and analysis provided to allow replication by others?

Yes

Reviewer Expertise:

Experiential learning, Ethnopharmacology, Toxicology, Pharmacy education, Therapeutics, Climate change and health, Public Health, Environmental Health.

I confirm that I have read this submission and believe that I have an appropriate level of expertise to confirm that it is of an acceptable scientific standard, however I have significant reservations, as outlined above.

# Response

**Published:** 2026-04-16

**Authors:** Aldizal Mahendra

Dear Dr Danladi Chiroma Husaini

 Thank you for your thoughtful and encouraging feedback. We have addressed your recommendations as follows:

1. Clarify research gap: The Introduction has been revised to explicitly state the novelty of combining multiple stakeholder perspectives in a single qualitative study.
2. Streamline introduction: Redundant sections have been merged for better flow and clarity.
3. Methods consistency: Duplicate text has been removed and methodological clarity improved.
4. Role of SEM in FGD design: Added explanation on how SEM informed question development.
5. Refinement of Table 2: Table 2 has been restructured to distinguish themes and sub-themes and align with SEM levels.
6. Integration of Figure 1: The figure is now explicitly discussed and linked to findings in the text.
7. Elevating discussion: We incorporated a higher-level analytical argument, emphasizing that unsafe practices are shaped by systemic constraints. Supporting participant quotes have been integrated.
8. Actionable recommendation: The Conclusion now includes a concrete pilot intervention model involving PKK, pharmacists, and local authorities.
9. Limitations refinement: Expanded to reflect contextual transferability across Indonesian regions.
10. Table 3 quote refinement: The less clear quote has been revised to improve clarity and contextual relevance.
11. Language and formatting: Comprehensive proofreading has been conducted.

We hope that these revisions have addressed the reviewer’s concerns and have strengthened the clarity, rigor, and overall contribution of the manuscript. We sincerely appreciate the reviewer’s insightful comments, which have been invaluable in improving the quality of this work.
